# FTH1 mRNA–Protein Discordance in Cardiac Ferroptosis: Multilayer Posttranscriptional and Degradative Control

**DOI:** 10.1155/cdr/8306021

**Published:** 2026-09-11

**Authors:** Feng Zhou, Jia-Bin Zhou, Yi-Qing Yan, Tian-Peng Wei, Dan Wu, Yi-Fan Yang, Ru-Xing Wang

**Affiliations:** ^1^ Department of Cardiology, Wuxi People′s Hospital Affiliated to Nanjing Medical University, Wuxi Medical Center, Nanjing Medical University, Wuxi, China, njmu.edu.cn

**Keywords:** cardiac ferroptosis, ferritinophagy, FTH1, iron homeostasis, IRP/IRE, posttranscriptional regulation

## Abstract

Ferritin heavy Chain 1 (FTH1) is a key protective factor against ferroptosis in the heart, yet its protein abundance can diverge from transcript levels in cardiac ferroptosis contexts. Since resistance to ferroptosis depends on FTH1 protein abundance rather than transcript levels and its ferroxidase activity cannot be readily compensated by other ferritin subunits, this mismatch is functionally consequential. This review organizes the evidence into three regulatory layers acting downstream of transcription. Iron regulatory protein/iron‐responsive element (IRP/IRE) signaling gates ribosome recruitment at the 5 ^′^‐untranslated region before translation begins. Epitranscriptomic marks and RNA‐binding proteins (RBPs) then route the transcript toward decay or translation, with the N^6^‐methyladenosine (m^6^A) reader YTHDF2 acting in opposite directions across cardiac contexts. Finally, nuclear receptor Coactivator 4 (NCOA4) delivers assembled ferritin for lysosomal degradation, depleting FTH1 protein independently of transcript level. Together, these mechanisms provide an integrated framework for understanding the mRNA–protein discordance of FTH1 and its contribution to cardiac ferroptosis susceptibility. We also discuss how this framework may inform layer‐specific therapeutic strategies and identify the measurements that are missing (labile iron pool, ferritin iron loading, and ferritinophagic flux) before mechanistic insight can translate into clinical cardioprotection.

## 1. Introduction

The heart is one of the most metabolically demanding organs, relying on oxidative phosphorylation to sustain continuous contractile function. This metabolic profile renders cardiomyocytes critically dependent on iron‐containing cofactors for mitochondrial respiration, oxygen sensing, and lipid metabolism [1]. Iron deficiency impairs mitochondrial energy reserve in cardiomyocytes, whereas iron overload catalyzes the production of reactive oxygen species (ROS) through the Fenton reaction, both leading to cardiac dysfunction [2, 3]. In the past decade, ferroptosis, an iron‐dependent form of regulated cell death characterized by the lethal accumulation of lipid hydroperoxides, has been increasingly recognized as a critical pathophysiological process in numerous cardiovascular diseases, including myocardial ischemia–reperfusion (I/R) injury, drug‐induced cardiomyopathy, heart failure, and diabetic cardiomyopathy [3–5].

Central to intracellular iron detoxification in cardiomyocytes is ferritin heavy Chain 1 (FTH1), a subunit with ferroxidase activity that oxidizes ferrous iron (Fe^2+^) to its ferric form (Fe^3+^), thereby enabling safe sequestration within the ferritin nanocage and limiting Fenton‐mediated radical generation [3, 5]. Genetic studies have established FTH1 as a nonredundant cardiac protector. Cardiomyocyte‐specific *Fth1* deletion causes iron‐dependent cardiomyopathy with hallmark features of ferroptosis [6], whereas disruption of nuclear receptor Coactivator 4 (NCOA4)–mediated ferritinophagy preserves ferritin and protects against cardiac dysfunction [7, 8]. This vulnerability is compounded by the subunit composition of cardiac ferritin: The H:L subunit ratio varies by tissue, with heart ferritin H‐subunit‐rich (H:L ≈ 1:1) and correspondingly high in ferroxidase activity [9, 10]. These findings position FTH1 not merely as an iron storage protein but as a critical determinant of ferroptosis susceptibility in the myocardium [4, 5].

The ability of FTH1 to buffer rapid fluctuations in the labile iron pool (LIP) demands regulatory mechanisms far faster than transcriptional induction. Ribosome profiling in human primary atrial cardiac fibroblasts revealed that *FTH1* translational efficiency can increase over threefold upon TGF*β*1 stimulation without changes in mRNA levels [11]. N^6^‐Methyladenosine (m^6^A)‐dependent pathways have been shown to either degrade *FTH1* mRNA [12] or promote its translation [2] in cardiomyocytes under ischemic stress, producing opposing effects on FTH1 protein output. In parallel, NCOA4‐mediated ferritinophagy can deplete FTH1 protein even when transcript levels are maintained or elevated [7, 8]. Each of these mechanisms uncouples protein from transcript, and none is visible to a transcriptome‐only analysis.

Despite growing recognition that FTH1 abundance is regulated at multiple levels beyond transcription, existing reviews have addressed IRP/IRE biology and ferritinophagy in a tissue‐general context [1, 7] or within the broader landscape of cardiac ferroptosis [3–5]. Neither treatment explains how these layers together uncouple FTH1 protein from its transcript in the heart. This gap is notable since these layers do not operate in isolation; rather, they may collectively set the threshold at which cardiomyocytes become vulnerable to ferroptosis.

We therefore focus on FTH1 mRNA–protein discordance as an integrating framework through which translational control, RNA regulation, and ferritinophagy converge across cardiac ferroptosis contexts. After summarizing FTH1 biology and its cardiac significance (Section 2), we examine how IRP/IRE signaling (Section 3), epitranscriptomic and RNA‐binding protein (RBP)–mediated regulation (Section 4), and NCOA4‐dependent ferritinophagy (Section 5) shape FTH1 abundance. Finally, we discuss how this framework may help distinguish where FTH1 loss arises from translational repression, aberrant RNA fate, or excessive ferritinophagic degradation, thereby informing mechanism‐based interventions (Sections 6 and 7). Every mechanism discussed is entered in Table 1 with its experimental system, evidence level, the nature of its link to FTH1, what was measured, and its principal limitation.

**Table 1:** Mechanisms regulating FTH1 in cardiac ferroptosis.

Mechanism	Cardiac system	Evidence level	FTH1 connection	Measured	m^6^A tier	Ref	Key limitation
Transcriptional input (Section 2.3)							
BACH1 represses *Fth1*/*Ftl1*/*Slc40a1* transcription	*Bach1* knockout mouse; acute myocardial infarction	Direct cardiac	Direct	mRNA, protein	—	[13]	Sets the input to the framework; transcriptional change alone cannot generate mRNA–protein discordance
NRF2 promotes *FTH1* transcription and sustains HERC2/VAMP8	None (MEFs and noncardiac lines; see Layer 3)	Extrapolated	Indirect	mRNA, protein, LIP, flux	—	[14, 15]	Dual position as transcriptional input and Layer 3 component; unconfirmed in heart
Ferritin function: Abundance is not equivalent to function (Section 2.4)							
PCBP1‐mediated iron loading of ferritin (abundance vs. function)	None (mammalian cell lines; purified proteins)	Extrapolated	Direct	Cited in this review via a general iron homeostasis reference	—	[1]	Ferritin iron loading changes without any change in total ferritin protein: The basis for treating FTH1 abundance as a proxy only. No cardiac test; no cardiac study has measured ferritin iron loading
Layer 1: IRP/IRE‐mediated translational repression (Section 3)							
Frataxin↓ → ISC↓ → IRP activation	Mouse ischemia–reperfusion (LAD); LV and whole‐heart homogenate; H9c2; HEK293T	Direct cardiac	Direct	Protein only (ferritin H)	—	[16]	No IRE‐binding assay performed; IRP1 and IRP2 protein both unchanged; activation inferred from cytosolic aconitase and target protein pattern; neither IRP manipulated; results and discussion give opposite ischemic phase directions
IRP2 stabilization by USP38	Mouse, STZ diabetes; cardiac‐specific USP38 transgenic and conditional knockout; left atrial tissue only; HL‐1; HEK293T	Direct cardiac	Direct	Protein only	—	[17]	Atrial only; ventricular relevance unconfirmed. IRP1 never measured; IRP2 never manipulated in vivo; *FTH1* mRNA not measured
IRP2 degradation by TRIM28 (K48‐linked ubiquitination at K877)	Mouse ischemia–reperfusion; *Trim28* cardiomyocyte‐inducible knockout; AAV9‐sh*Irp2*; NRVM; HEK293T; AMCM (iron content only); human ischemic heart disease explants (*n* = 5) vs. donor (*n* = 3)	Direct cardiac	Indirect	Ferritin not measured; TFR1 mRNA and protein; total iron	—	[18]	This study measured neither FTH1 nor FTL; the consequence for *Fth1* translation is inferred from the IRP2–IRE mechanism. Single study; human arm is *n* = 5 vs. 3, association only, without ferritin
p53/DJ‐1 (PARK7) → ISC disruption → IRP2 accumulation	Mouse, chronic doxorubicin; cardiomyocyte‐specific *Trp53* knockout; AAV9‐cTnT‐*Park7*; AMCM (Langendorff); NMCM	Direct cardiac	Direct	Protein only (ferritin H)	—	[19]	IRP1 explicitly unchanged; neither IRP was manipulated, so the chain ends at IRP2 accumulation. Ferritin H direction is preparation‐dependent (up in AMCM, down in NMCM); doxorubicin‐specific
ISC sensing by IRP2 independent of IRP2 protein level	None (breast and lung cancer lines, HEK293; 3% O_2_)	Extrapolated	Direct	mRNA and protein, same experiment—Discordant	—	[20]	No cardiac validation. The cleanest within‐experiment *FTH1* mRNA–protein discordance available, but the FBXL5‐independent mechanism appears only at 3% O_2_, whereas cardiac studies culture at 21%
*Irp1*/*Irp2* double knockout → cardiac iron deficiency	Mouse; cardiomyocyte‐targeted double floxed knockout; isolated adult cardiomyocytes; dobutamine stress; LAD myocardial infarction; human LV explants (ICM/DCM) vs. donor	Direct cardiac	Direct	Protein only (ferritin H, 249*%* ± 35*%*); cardiomyocyte iron; IRE‐binding EMSA	—	[21]	Double knockout; single‐gene contributions unclear. Phenotype is iron deficiency, not ferroptosis: FTH1 rose, whereas myocyte iron fell. Human EMSA measures total IRP activity (no supershift), *n* = 4–6
IRP2 overexpression → paradoxical ferritin H↑	None (mouse substantia nigra; PC‐12)	Extrapolated	Direct	mRNA (microarray) and protein—Discordant, but different experiments	—	[22]	No cardiac validation. A counterexample to the simple IRP2↑ → FTH1↓ rule, as the authors state; mRNA and protein come from separate datasets
Layer 2: Epitranscriptomic, RNA‐binding protein, and noncoding RNA regulation (Section 4)							
Lactate → H3K18la → YTHDF2↑ → *FTH1* mRNA decay	Mouse ischemia–reperfusion (LAD); intramyocardial shRNA; H9c2 only	Direct cardiac	Direct	(i) + (ii) + (iii)	mRNA and protein—Concordant	[12]	The only cardiac study reaching all three tiers. m^6^A evidence is bulk MeRIP‐qPCR at computationally predicted sites, not single‐nucleotide resolution; no wild‐type vs. m^6^A‐mutant reporter; H9c2 only
ALKBH5 → YTHDF1↑ → *Fth1* translation↑	Rat myocardial ischemia–reperfusion (Sprague–Dawley); tail‐vein lentivirus; H9c2 only	Direct cardiac	Indirect	(iv)	mRNA unchanged, protein changed—Discordant	[2]	ALKBH5 acts on *Ythdf1*, not *Fth1*; MeRIP was performed on *Ythdf1*, and the reader‐binding RIP used an anti‐FTH1 antibody, which cannot show YTHDF1 occupancy. No polysome profiling or reporter
METTL3 → m^6^A‐*NCOA4* → YTHDF2 → NCOA4↓ (FTH1 preserved)	Mouse myocardial infarction; AAV9‐YTHDF2; HL‐1; H9c2; NMCM; AMCM (YTHDF2 localization only)	Direct cardiac	Indirect	(iv)	mRNA and protein—Concordant	[23]	m^6^A‐seq was global; RIP and decay assays addressed *NCOA4* only, so no head‐to‐head comparison of YTHDF2 occupancy on *FTH1* vs. *NCOA4* exists. Assigns YTHDF2 the opposite net role to [10]
HnRNPA2B1 → m^6^A‐*PFN2* stabilization → FTH1↓	*Hnrnpa2b1* knockout mouse ischemia–reperfusion; Sprague–Dawley rat; NRVM; H9c2; human serum (*n* = 32 vs. 35)	Direct cardiac	Indirect	(iv)	Protein only	[24]	All m^6^A, RIP, and mutant reporter work addressed *PFN2*; FTH1 lies two steps downstream with no direct linkage. *Fth1* transcript never measured. Human data are serum, not myocardium
HNRNPC knockdown → FTH1 protein preserved	Mouse, Angiotensin II osmotic minipump, 4 weeks; AC16 only	Cardiac‐associated	Correlative	(iv)	Protein only	[25]	No m^6^A assay of any kind and no binding assay; intermediaries undefined. The FTH1 response is biphasic (up over the first 6 h, then down), so the axis is not simple FTH1 suppression
METTL14 → m^6^A‐*FTH1* → decay	None (cervical cancer lines; 53 paired human cervical tissues; nude‐mouse xenograft)	Extrapolated	Direct	(i) + (iii), with wild‐type/mutant 3 ^′^‐UTR reporter	mRNA and protein—Concordant	[26]	No cardiac validation. m^6^A evidence is bulk MeRIP‐qPCR, not single‐nucleotide; the reporter is writer‐driven, with no reader RIP or CLIP
IGF2BP1 (lncRNA *CACNA1G-AS1* axis) → *FTH1* stabilization	None (ovarian cancer lines; 124 tumor vs. 56 normal ovarian tissues; nude‐mouse xenograft)	Extrapolated	Indirect	(iv) + (iii)	mRNA and protein—Concordant	[27]	The authors state explicitly that *FTH1* methylation was not measured; the immunoprecipitation used an anti‐AGO2 rather than an anti‐IGF2BP1 antibody, so the link is epistatic. Not examined in heart
NSUN5 → m^5^C → TRAP1 → FTH1/FTL expression↑	None (rat bone marrow–derived mesenchymal stem cells; erastin)	Extrapolated	Direct	Not an m^6^A study; m^5^C mapped at single‐nucleotide resolution	m^5^C on endogenous *FTH1* (5 ^′^‐UTR) and *FTL* (3 ^′^‐UTR) by bisulfite sequencing; protein by immunoblot; no mRNA‐stability assay	[28]	No cardiac validation. TRAP1 recruitment shown by coimmunoprecipitation, but the paper demonstrates m^5^C → protein expression, not a measured mRNA half‐life
ELAVL1/HuR → BECN1 → ferritinophagy	None (hepatic stellate cells)	Extrapolated	Indirect	(iv)	—	[29]	BECN1 mechanism not confirmed in heart; FTH1 not assayed
ELAVL1/HuR proferroptotic in cardiac ischemia–reperfusion	Mouse ischemia–reperfusion; HCM (human cardiomyocyte line)	Cardiac‐associated	Indirect	(iv)—Not an m^6^A study	Protein only, in vivo only	[30]	RIP, biotin pull‐down, and luciferase all addressed *Becn1* and the *ELAVL1* promoter, never *FTH1*. No effect of ELAVL1 manipulation on FTH1 was reported; supports only that FTH1 protein falls in murine ischemia–reperfusion
FTO demethylates → HuR stabilizes *Trp53*/*Cdkn1a*/*Nfe2l2* → antiferroptotic	Mouse doxorubicin; AAV9‐FTO; H9c2; AC16 (p53/p21 dose–response only)	Direct cardiac	Indirect	(iv)	Protein only	[31]	MeRIP and HuR RIP addressed *Trp53*/*Cdkn1a*/*Nfe2l2*; no assay of any kind on *FTH1*, which is only a downstream NRF2‐target readout. Dual role with the BECN1 pathway; net effect is context‐dependent
*CircSnx12*/miR‐224‐5p → *FTH1* 3 ^′^‐UTR repression	Mouse transverse aortic constriction; HL‐1 only (luciferase)	Direct cardiac	Direct	n/a (miRNA)	mRNA and protein—Concordant	[32]	Direct targeting rests only on a wild‐type vs. mutant 3 ^′^‐UTR luciferase reporter; no AGO2 RIP, RNA pull‐down, or *circSnx12* gain/loss of function, so the circRNA arm is inferred. Results and limitations disagree on whether *circSnx12* was measured
Layer 3: NCOA4‐mediated ferritinophagy (Section 5)							
NCOA4 as cargo receptor; FTH1 R23 recognition; whole‐cage targeting	None (293T, U2OS, HCT116, K562; recombinant ferritins; zebrafish)	Extrapolated	Direct	Protein; flux (E64d/pepstatin and bafilomycin clamps, lysosomal ferritin puncta, NCOA4–FTH1 coimmunoprecipitation, direct binding)	—	[33, 34]	Foundational, but no cardiac system in either study. Recognition requires FTH1 R23, yet one or a few wild‐type subunits suffice to target an entire 24‐mer, so FTL is codelivered; FTL degradation was not tracked
HERC2 → NCOA4 ubiquitination (iron‐dependent)	None (293T, U2OS; recombinant protein and ICP‐MS)	Extrapolated	Direct	Protein; NCOA4 cycloheximide chase	—	[34]	Direct HERC2–NCOA4 regulation has not been demonstrated in any cardiac system
ATM → NCOA4 S550 phosphorylation	None (MEFs ± *Atm*/*Trp53*; HT‐1080; HCT116 *TP53*−/−)	Extrapolated	Indirect	Protein; flux (chloroquine clamp on FTH1 degradation, FTH1–LAMP1 colocalization, NCOA4–FTH1 coimmunoprecipitation, ATG9/VPS34 rescue)	—	[35]	No cardiac validation and no in vivo model. An FTL antibody is listed, but FTL is never reported as data
SENP2↓ → NCOA4 SUMOylation↑ → OTUB1‐mediated stabilization	Mouse ischemia–reperfusion; global *Senp2* knockout plus cardiac SENP2 transgenic; AC16 only; HEK293T	Direct cardiac	Indirect	Protein; partial flux (chloroquine clamp on LC3/SQSTM1; FTH1–LAMP1 colocalization)	—	[36]	Single study. *Senp2* knockout is global, not cardiac‐restricted; NCOA4 was never knocked down; no NCOA4–FTH1 coimmunoprecipitation; the clamp was not applied to the ferritin readout. FTL not measured
Cardiac‐specific *Ncoa4* knockout → FTH1 preserved	Mouse transverse aortic constriction; cardiomyocyte‐specific *Ncoa4* knockout (Myh6‐Cre with Cre‐only control); isolated adult mouse ventricular cardiomyocytes	Direct cardiac	Direct	mRNA and protein—Discordant (protein↓ from d5, *Fth1* mRNA↑ d5–7); LIP; partial flux (FTH1–LC3B and FTH1–LAMP2a colocalization with genetic rescue)	—	[8]	Loss of function only; no inhibitor clamp, so flux is inferred from static colocalization plus genetic rescue. Discordance is confined to the early phase; by 4 weeks, *Fth1* mRNA had also fallen. Cardiac FTL not measured
STAT3 → *Ncoa4* transcription↑	*Apoe*−/− mouse, 12‐week high‐fat diet; piperlongumine; H9c2; AC16	Direct cardiac	Indirect	Protein; flux—The strongest in the cardiac set (bafilomycin A1 vs. 3‐MA clamp applied directly to FTH1; mRFP‐GFP‐LC3B tandem reporter; FTH1–LC3 colocalization; NCOA4 siRNA rescue)	—	[37]	Obesity‐specific and on an *Apoe*−/− background. No binding assay of any kind; the in vivo arm reports steady‐state abundance only. FTL not measured
DNMT1 → NCOA4 upregulation	Sprague–Dawley rat, STZ diabetes plus ischemia–reperfusion; 5‐aza‐CdR; H9c2 only; no transgenic animal	Direct cardiac	Indirect	Protein only—Steady‐state abundance, no flux assay	—	[38]	Diabetic ischemia–reperfusion specific. No clamp, reporter, coimmunoprecipitation, ATG manipulation, or cycloheximide chase. Beclin‐1 and p62 were unchanged, and the TEM difference was not significant. FTL never mentioned
Lipopolysaccharide → NCOA4 → ferritin↓	Mouse lipopolysaccharide septic cardiomyopathy; AAV9‐sh*Ncoa4*, not cell‐type‐targeted; H9c2 only (described as myofibroblasts, partly ATRA‐differentiated)	Direct cardiac	Direct	Protein; partial flux (ferritin–NCOA4 coimmunoprecipitation; 3‐MA and *Atg5* siRNA rescue; MG132 specificity control)	—	[39]	Upstream NCOA4 activator undefined. LC3 and p62 were never measured, and no lysosomal clamp was applied. The immunoblot antibody is listed only as “ferritin,” so the subunit assayed is unspecified
PM_2.5_ → ferritinophagy → cardiac hypertrophy	Mouse intratracheal PM_2.5_, 4 weeks; no in vivo genetic manipulation; AC16 only (stable sh*NCOA4*)	Direct cardiac	Direct	Protein only—Steady‐state abundance	—	[40]	Environmental exposure model; clinical relevance uncertain. No flux assay: LC3‐II and p62 rose together without a clamp, a pattern equally consistent with blocked flux. FTH1 is biphasic. FTL not measured
NRF2 → HERC2 and VAMP8 (multilayer)	None (ovarian cancer lines, patient‐derived lines; NSG xenograft/PDX)	Extrapolated	Indirect	mRNA and protein—Discordant (FTH1/FTL protein↑ despite mRNA↓); LIP; flux (mRFP‐GFP‐LC3 tandem reporter, TEM, quantified FTH1/FTL–LC3 vs. FTH1/FTL–LAMP1 colocalization, rapamycin rescue)	—	[14, 15]	Integrated coordinator unconfirmed in heart. The only study here that tracks FTL as cargo, and the clearest case that high FTH1 protein can coexist with failed iron handling (apoferritin trapped in unfused autophagosomes)

*Note:* Evidence level. *Direct cardiac*, demonstrated in cardiac tissue or cardiac cells; *cardiac-associated*, cardiac study but the mechanistic link to FTH1 is incomplete; *extrapolated*, rests on noncardiac systems and awaits cardiac validation. FTH1 connection. *Direct*, the regulator acts on *FTH1* mRNA or FTH1 protein itself; *indirect*, acts through an intermediary transcript or protein; *correlative*, only an association was reported. Measured. What the study assayed for ferritin. Where transcript and protein were both measured in the same experiment, the entry states whether they moved concordantly or discordantly. m^6^A evidence tier: (i) m^6^A mapped on endogenous *FTH1* mRNA, (ii) reader binding to *FTH1* mRNA demonstrated, (iii) functional epistasis with FTH1, and (iv) indirect inference only. Applies to Layer 2 entries.

Abbreviations: AAV9, adeno‐associated virus Serotype 9; AC16, human cardiomyocyte line; AMCM, adult mouse cardiomyocytes; Ang II, Angiotensin II; ATM, ataxia‐telangiectasia mutated; DOX, doxorubicin; HF, heart failure; I/R, ischemia–reperfusion; IRE, iron‐responsive element; IRP, iron regulatory protein; ISC, iron–sulfur cluster; LIP, labile iron pool; MeRIP, methylated RNA immunoprecipitation; MI, myocardial infarction; NRVM, neonatal rat ventricular myocyte; RIP, RNA immunoprecipitation; TAC, transverse aortic constriction.

This is a narrative review assembled by a thematically structured search rather than a pre‐registered protocol. We searched PubMed from database inception to April 2026, in successive rounds organized around the regulatory mechanisms covered by this review. Terms for FTH1 and ferritin (FTH1, ferritin heavy chain, and H‐ferritin) were combined with mechanism terms and with cardiac terms (heart, cardiac, cardiomyocyte, myocardial, I/R, cardiomyopathy, heart failure, and atrial fibrillation). Titles and abstracts were screened first, full texts retrieved for candidate studies, and reference lists of retrieved articles screened manually. We included primary studies reporting a mechanistic link between a regulator and *FTH1* mRNA fate or FTH1 protein abundance and prioritized work in cardiac tissue or cardiac cells. Noncardiac studies were retained only where they establish a mechanism unavailable in cardiac systems but needed for the framework to cohere. Since the cardiac literature at several nodes consists of single studies, we did not attempt a systematic review or meta‐analysis and make no claim of exhaustive coverage.

## 2. FTH1: Structure, Ferroxidase Activity, and Cardiac Significance

### 2.1. Architecture of the Ferritin Nanocage and FTH1 Ferroxidase Function

Mammalian cytosolic ferritin assembles as a 24‐subunit heteropolymer of heavy (FTH1, ~21 kDa) and light (FTL, ~19 kDa) chains, forming a hollow nanocage capable of storing up to 4500 iron atoms as a nontoxic ferrihydrite mineral core [9]. Functionally, FTH1 carries a diiron ferroxidase center that oxidizes Fe^2+^ to Fe^3+^ as the initiating step of mineralization, whereas FTL lacks ferroxidase activity and provides nucleation sites for growth of the mineral core [9, 10]. The ferroxidase reaction consumes Fe^2+^ together with O_2_ or H_2_O_2_, with the relative contribution of each oxidant depending on iron loading and reaction conditions [9]. In either case, FTH1 limits radical generation principally by oxidizing Fe^2+^ and withdrawing it from the LIP, rather than by scavenging peroxide directly [9, 10]. FTH1 thus catalyzes rapid iron detoxification, whereas FTL supports stable storage. The FTH1‐to‐FTL ratio varies by tissue: Liver and spleen ferritin is FTL‐enriched for long‐term storage, whereas heart, brain, and kidney ferritin contains a higher proportion of FTH1, reflecting the demand for rapid iron detoxification in metabolically active organs [9, 10].

### 2.2. FTH1 as a Nonredundant Cardiac Protector Against Ferroptosis

FTH1, with its ferroxidase activity, serves as the primary enzymatic barrier between the cytosolic LIP and Fenton‐mediated lipid peroxidation. Its indispensability is directly demonstrated by genetic loss‐of‐function and pathway‐perturbation studies. Cardiomyocyte‐specific *Fth1* knockout mice developed iron‐dependent cardiomyopathy with hallmark features of ferroptosis. The phenotype was fully rescued by ferrostatin‐1 and by overexpression of the cystine transporter *Slc7a11*, positioning FTH1 loss as a key determinant that sensitizes the heart to ferroptotic death [6]. Conversely, disruption of NCOA4‐mediated ferritinophagy attenuated ferritin degradation and protected against cardiac dysfunction, demonstrating that the balance between ferritin synthesis and degradation determines cardiac ferroptosis susceptibility [7].

### 2.3. Defining FTH1 mRNA–Protein Discordance

In this review, discordance means that *FTH1* mRNA is an unreliable readout of FTH1 protein within the same sample set: The two move in opposite directions, or protein changes whereas the transcript does not. Where transcript and protein fall together, as in m^6^A‐dependent decay of *FTH1* mRNA, the regulation is posttranscriptional but concordant, and falls outside this definition.

Transcriptional regulation determines the size of the *FTH1* mRNA pool available to the three posttranscriptional and degradative layers, but it does not by itself generate discordance. Transcription is therefore treated as the input to the framework rather than as a fourth layer. BACH1, a heme‐responsive transcriptional repressor, directly suppresses *Fth1*, *Ftl1*, and *Slc40a1* transcription; *Bach1* knockout mice exhibited smaller infarct size and reduced ferroptosis after acute myocardial infarction [13]. Thus, BACH1 establishes a transcriptional floor beneath which even maximal translational derepression cannot compensate. NRF2 sets the opposing bound by driving *FTH1* transcription. It is also the only regulator that acts within a layer as well as at the input, since it sustains HERC2‐dependent NCOA4 turnover (Section 5.1.3). The layers examined in Sections 3, 4, and 5 act on transcripts that have already been made.

### 2.4. FTH1 Abundance Is Not Equivalent to Ferritin Function

A cell can accumulate FTH1 protein and still fail to sequester iron. In NRF2‐deficient cells, FTH1 and FTL proteins rise despite falling transcript levels, yet the LIP expands, since iron‐free apoferritin accumulates in autophagosomes that cannot fuse with lysosomes [14]. Protein abundance and iron‐buffering capacity are therefore related but nonidentical quantities, and three factors separate them. First, ferroxidase capacity depends on the H:L stoichiometry of the individual 24‐mer rather than on total FTH1: In reconstituted human ferritins, H‐rich and L‐rich cages differ in both oxidation kinetics and mineral core size, and the L subunit modulates turnover at the H‐subunit ferroxidase center [9, 10]. Second, ferritin function depends on the iron loading state of the cage, set in part by cytosolic iron chaperones such as PCBP1 that deliver iron to ferritin largely independently of ferritin protein abundance [1]. Apoferritin and iron‐loaded holoferritin are indistinguishable by immunoblot, yet only the latter represents sequestered iron. Third, the ferroxidase reaction does not have a single fixed relationship to peroxide (Section 2.1).

The limitation is directional. A fall in FTH1 protein reliably indicates reduced buffering capacity, since ferroxidase activity cannot be sustained without the subunit that carries it, and cardiomyocyte‐specific *Fth1* deletion alone is sufficient to cause ferroptotic cardiomyopathy (Section 2.2). A rise cannot be read the same way: An intervention that preserves FTH1 protein without restoring iron sequestration would register as a success on immunoblot while leaving the LIP expanded (Section 6.2). Table 1 records, for each study, whether FTH1 protein was corroborated by a direct iron measurement.

## 3. The IRP/IRE System: Translational Gatekeeping of FTH1 in the Heart

### 3.1. Classical Architecture of the IRP/IRE Regulatory Axis

The posttranscriptional control of FTH1 is anchored by a highly conserved iron‐responsive element (IRE), a ~28–30‐nucleotide stem‐loop in the 5 ^′^‐UTR of *FTH1* mRNA that is both necessary and sufficient to confer translational regulation by iron [41, 42]. The position of the element within the transcript dictates the mode of control: A 5 ^′^‐UTR IRE governs translation, whereas 3 ^′^‐UTR IREs govern mRNA stability [43]. Two trans‐acting factors decode this signal. Iron regulatory Protein 1 (IRP1, encoded by *ACO1*) functions as cytosolic aconitase when assembled with a [4Fe‐4S] cluster under iron‐replete conditions. When iron or iron–sulfur cluster (ISC) availability diminishes, IRP1 loses this cluster and switches to a high‐affinity IRE‐binding conformation, making it a molecular toggle directly coupled to iron and ISC status [1, 3, 42]. Iron regulatory Protein 2 (IRP2, encoded by *IREB2*) lacks aconitase activity and is classically regulated through FBXL5‐dependent ubiquitination and proteasomal degradation under iron‐replete conditions [20, 44].

When either IRP binds the 5 ^′^‐UTR IRE of *FTH1* mRNA, it prevents recruitment of the 43S preinitiation complex and the small ribosomal subunit, blocking translation initiation [42, 45, 46]. The net outcome is a decrease in FTH1 protein with unchanged mRNA, providing a canonical mechanism for the mRNA–protein discordance [20, 47]. Animal models reveal that the two IRPs are nonredundant but compensatory: Tissue‐specific double knockouts are lethal, and IRP2 generally dominates iron homeostasis in most tissues, whereas IRP1 serves as a backup sensor under oxidative stress or ISC‐deficient conditions [47, 48]. This issue becomes particularly relevant in the disease settings discussed below.

### 3.2. IRP/IRE‐Mediated Translational Control Dominates FTH1 Protein Output in the Heart

The hemojuvelin knockout model introduced in Section 2.3 provided the first systematic indication that translational control may dominate FTH1 regulation in the heart. Brewer et al. [49] observed in whole‐heart homogenate that cardiac *Tfr1* transcript was inversely correlated with heart iron concentration, whereas *Dmt1* showed a paradoxical positive correlation that the authors noted was inconsistent with classical IRE/IRP regulation, whereas *Fth1* (reported in the original study as H‐ferritin mRNA) was constitutively expressed regardless of cardiac iron burden. Since IRP/IRE‐mediated regulation operates at the translational level without altering mRNA abundance, the authors reasoned that this transcript‐level pattern, iron‐responsive *Ftl1* alongside iron‐invariant *Fth1*, is most consistent with IRP/IRE‐dependent translational control of FTH1 in the heart [49].

The study by Haddad et al. [21] provided direct genetic evidence that IRP function is indispensable for cardiac health. Using cardiomyocyte‐targeted deletion of both *Irp1* and *Irp2*, the investigators generated mice with cardiac‐specific functional iron deficiency in the absence of systemic anemia. Transferrin Receptor 1 (TFR1) was downregulated to 25% of control levels, whereas ferroportin (325%) and FTH1 (ferritin H‐chain; 249%) were markedly upregulated, a pattern fully consistent with loss of IRP‐mediated translational repression of FTH1 and stabilization of *Tfr1* mRNA [21]. The resulting FTH1 derepression was accompanied by mitochondrial dysfunction and impaired stress tolerance, illustrating that IRP loss simultaneously disrupts both iron sequestration and iron supply in the heart [21]. Crucially, single‐gene knockouts of either *Irp1* or *Irp2* alone did not produce an overt cardiac phenotype, indicating substantial functional compensation between the two proteins in the heart under basal conditions [21].

Together, these two studies provide complementary support: Brewer shows the transcript‐level signature expected of IRP/IRE dominance during iron overload, whereas Haddad reveals the functional consequences of IRP loss. It is important to note that the Haddad phenotype is one of cardiac iron deficiency, not iron overload: Removing IRP‐mediated repression raised FTH1 protein and lowered cardiomyocyte iron, impairing mitochondrial respiration and stress tolerance. The two studies therefore bracket the IRP/IRE system from opposite directions. The convergence of these gain‐of‐iron and loss‐of‐IRP models supports the view that IRP/IRE‐mediated translational control is a major mechanistic basis for FTH1 mRNA–protein divergence in the heart.

### 3.3. IRP/IRE Dysregulation as a Driver of Cardiac Ferroptosis

#### 3.3.1. Frataxin Loss and ISC Disruption: IRP‐Dependent FTH1 Suppression

IRP1‐mediated ferroptosis was first characterized in cancer cells, where erastin promotes aconitase/IRP1 conversion [48, 50]. In the heart, Zhang et al. [16] reported that frataxin, essential for ISC biosynthesis, is upregulated during myocardial ischemia and suppressed upon reperfusion. During reperfusion, frataxin decline leads to reduced ISC availability, enhanced IRP binding activity, and a characteristic FTH1‐down/TFR1‐up phenotype with ferroptotic cardiomyocyte death. Adeno‐associated virus Serotype 9 (AAV9)–mediated frataxin overexpression restored IRP aconitase activity and reduced infarct size [16].

**Figure 1:**
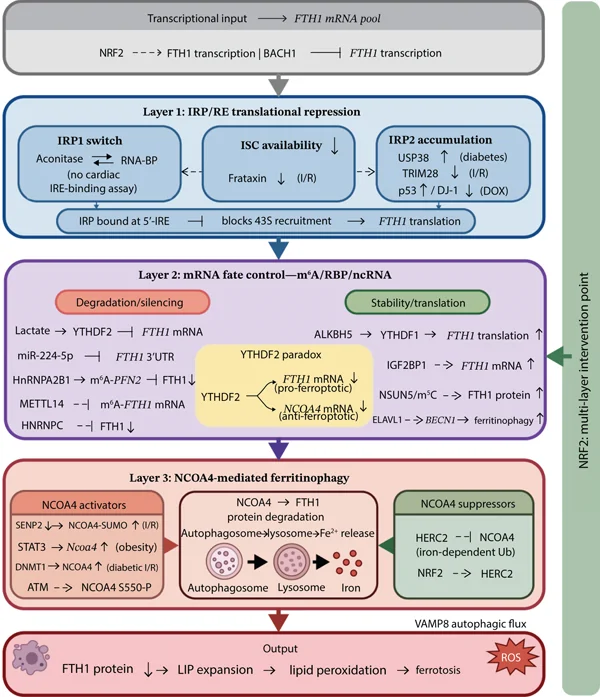
Multilayer posttranscriptional and degradative regulation of FTH1 in cardiac ferroptosis. Transcriptional input sets the FTH1 mRNA pool; three layers then act downstream of it. Layer 1, IRP/IRE‐mediated translational gating at the 5 ^′^‐IRE. Layer 2, control of transcript fate by m^6^A, RBPs, and noncoding RNAs. Layer 3, NCOA4‐mediated ferritinophagy, in which recognition of R23 on FTH1 delivers the intact 24‐mer, so FTL is codegraded. NRF2 (green sidebar) acts at more than one level. Line style denotes the level of cardiac evidence, as classified in Table 1: solid, demonstrated in cardiac tissue or cardiac cells; dashed, extrapolated from noncardiac systems; dotted, cardiac‐associated. Arrowheads denote activation; bars denote inhibition. DOX, doxorubicin; IRE, iron‐responsive element; IRP, iron regulatory protein; I/R, ischemia–reperfusion; ISC, iron–sulfur cluster; LIP, labile iron pool; m^5^C, 5‐methylcytosine; m^6^A, N^6^‐methyladenosine; RBP, RNA‐binding protein.

Aconitase activity reports IRP1, but ISC depletion is not an IRP1‐specific input: It converts IRP1 to its RNA‐binding form and independently enhances IRP2 IRE binding without altering IRP2 protein level [20]. In the cardiac study, IRP activation was tracked by cytosolic aconitase activity, which reports IRP1 only, with no IRE‐binding assay to resolve the two [16]. Frataxin loss therefore belongs at the shared ISC‐sensing node upstream of both IRPs (Figure 1) rather than assigned to IRP1 alone. In either case, the downstream consequence converges on FTH1: Enhanced IRP binding to the 5 ^′^‐UTR IRE suppresses *Fth1* translation, depletes the ferroxidase‐competent iron buffer, and expands the LIP toward the ferroptotic threshold.

#### 3.3.2. IRP2 Accumulation: Converging Pathways to *FTH1* Translational Suppression

Beyond ISC‐dependent enhancement of IRP2 binding activity, which has been demonstrated in noncardiac models but awaits cardiac validation [20], several recent studies have identified cardiac‐specific regulators of IRP2 turnover that converge on *FTH1* translational suppression. Xiao et al. [17] found that USP38 stabilizes IRP2 in diabetic atrial cardiomyocytes, enhancing IRP2‐mediated *Fth1* translational repression and leading to iron overload and atrial fibrillation susceptibility. Conversely, Zhu et al. [18] identified TRIM28 as a cardiac E3 ubiquitin ligase that promotes K48‐linked ubiquitination of IRP2 at K877. During I/R injury, TRIM28 downregulation leads to IRP2 accumulation, raising TFR1 and cardiac iron and driving cardiomyocyte ferroptosis. FTH1 itself was not measured, and the consequence for *Fth1* translation follows from the IRP2–IRE mechanism (Table 1). Human myocardium supports the axis: IRP2 and TFR1 proteins were elevated in ischemic and border zone tissue from explanted ischemic hearts, with no difference in remote myocardium [18].

The p53/PARK7 axis adds another layer. In doxorubicin (DOX) cardiotoxicity, p53 activation drives PARK7 degradation, compromising ISC biosynthesis and allowing IRP2 to accumulate. *Park7* knockdown raised IRP2 further, and *Park7* overexpression reversed the rise, whereas IRP1 protein was unchanged throughout [19]. The pathway is therefore IRP2‐associated rather than IRP1‐associated (Figure 1).

#### 3.3.3. The IRP1 Versus IRP2 Question: Which Drives FTH1 Suppression?

An unresolved issue is which IRP dominates iron sensing in the diseased heart. Genetic data from Haddad et al. [21] suggest extensive compensation under basal conditions, but pathological stressors may shift this balance. IRP1 activation is favored when ISC assembly is acutely disrupted, although in the cardiac frataxin study, this was inferred from aconitase activity rather than demonstrated by isoform‐resolved IRE binding [16] (Section 3.3.1). IRP2 accumulation predominates when its ubiquitin‐dependent clearance is impaired (e.g., USP38 stabilization in diabetes [17] and TRIM28 loss in I/R [18]). Whether compensatory dynamics can override IRP2‐mediated repression in the heart, as suggested by paradoxical FTH1 upregulation upon IRP2 overexpression in neural cells [22], remains untested. Resolving the IRP1‐versus‐IRP2 hierarchy in cardiac ferroptosis contexts will require simultaneous measurement of *FTH1* mRNA, translational efficiency, and protein turnover within the same injury model.

## 4. Epitranscriptomic, RBP, and Noncoding RNA (NcRNA)–Mediated Regulation of FTH1

The IRP/IRE system provides translational gatekeeping of FTH1 in direct response to iron and ISC availability. However, the heart is simultaneously exposed to noniron metabolic stresses, such as ischemic lactate accumulation, oxidative bursts, and neurohormonal activation, which do not signal through the LIP. Epitranscriptomic modifications and RBPs have emerged as a second posttranscriptional layer that integrates these diverse metabolic cues into *FTH1* mRNA fate decisions [51]. Whereas IRP binding responds within minutes to changes in iron availability, RBP recruitment and epitranscriptomic remodeling unfold over hours, providing a slower but sustained regulatory layer particularly relevant during chronic or subacute cardiac injury [52]. In this framework, m^6^A modification functions as a signal‐responsive routing mechanism that directs *FTH1* mRNA toward either stabilization and translation or degradation, depending on which writers, erasers, and readers are activated by the prevailing pathological context.

### 4.1. m6A Modification as a Context‐Dependent Determinant of *FTH1* mRNA Fate

Among these epitranscriptomic mechanisms, m^6^A modification has received growing attention in the context of cardiac ferroptosis [52]. m^6^A is dynamically installed by methyltransferases (METTL3, METTL14, and WTAP), removed by demethylases (ALKBH5 and FTO), and interpreted by reader proteins (YTHDF1/2/3, YTHDC1/2, IGF2BP1/2/3, HNRNPC, and hnRNPA2B1) [52]. A defining feature of m^6^A‐mediated regulation is its high context dependence: The same m^6^A mark on the same transcript can trigger degradation or promote translation depending on which reader is recruited, which in turn is determined by the cellular stress environment. In cardiac ferroptosis contexts, multiple distinct m^6^A‐dependent pathways converge on FTH1, with opposing functional outcomes.

#### 4.1.1. YTHDF2‐Mediated *FTH1* mRNA Degradation

The most direct evidence for m^6^A‐dependent FTH1 regulation in the heart comes from Xiang et al. [12], who demonstrated that during myocardial I/R injury, ischemia‐induced lactate accumulation drives histone H3 lysine 18 lactylation (H3K18la) at the YTHDF2 promoter, transcriptionally upregulating YTHDF2. The increased YTHDF2 then binds m^6^A‐modified *FTH1* mRNA and accelerates its degradation, depleting FTH1 protein and triggering cardiomyocyte ferroptosis. In H9c2 cardiomyoblasts, YTHDF2 knockdown stabilized *FTH1* mRNA and reduced infarct size. Critically, simultaneous FTH1 silencing abolished the protection, confirming that FTH1 is a functionally essential downstream target of YTHDF2 in this model [12]. This links ischemia‐associated lactate accumulation directly to epitranscriptomic control of cardiac iron handling, a form of metabolite‐to‐RNA signaling that bypasses the canonical IRP/IRE circuit.

#### 4.1.2. The ALKBH5/YTHDF1 Axis Promotes FTH1 Translation

In a contrasting protective pathway during myocardial I/R injury, Yin et al. [2] showed that ALKBH5 overexpression upregulated FTH1 protein without altering its mRNA, a hallmark of translational regulation. Mechanistically, ALKBH5 demethylated *Ythdf1* mRNA, increasing YTHDF1 protein levels, which promoted *Fth1* translation. In vivo, this axis reduced infarct size and fibrosis [2]. The regulation is indirect, and its final step is inferred: ALKBH5 demethylates *Ythdf1* rather than *Fth1*, and no assay in that study places YTHDF1 on the *Fth1* transcript [2].

#### 4.1.3. The YTHDF2 Paradox: Direct Degradation Versus Indirect Preservation

YTHDF2 appears to play opposite roles in different cardiac injury settings. As described above, Xiang et al. [12] demonstrated that YTHDF2 directly degrades *FTH1* mRNA during I/R injury, promoting ferroptosis. However, in a myocardial infarction model, Shao et al. [23] reported that METTL3‐mediated m^6^A modification of *NCOA4* mRNA recruits YTHDF2, which accelerates NCOA4 degradation. Since NCOA4 is the cargo receptor for ferritinophagy (Section 5), YTHDF2‐dependent depletion of NCOA4 suppresses ferritin autophagic clearance. In that model, YTHDF2 overexpression restored FTH1 at both the transcript and protein levels and was net cardioprotective [23]. The same reader therefore protects or depletes FTH1 depending on which transcript it engages, and global inhibition of YTHDF2 would stabilize *FTH1* mRNA while simultaneously derepressing NCOA4‐mediated ferritinophagy, with unpredictable net effects on cardiomyocyte iron handling (Section 6.2).

We propose, as a working hypothesis rather than an established mechanism, that this divergence reflects transcript selection, with the net outcome of YTHDF2 activation set by the relative abundance and m^6^A density of competing transcripts under distinct pathological conditions. During acute I/R with severe lactate accumulation, YTHDF2 upregulation may be sufficiently robust to deplete *FTH1* mRNA directly [12]. In contrast, during myocardial infarction, the inflammatory and autophagic response may predominate. However, no cardiac study has compared YTHDF2 occupancy on *FTH1* and *NCOA4*, and no m^6^A site on *FTH1* has been mapped at single‐nucleotide resolution in any cardiac system. Testing the hypothesis requires parallel YTHDF2 RNA immunoprecipitation (RIP) or cross‐linking and immunoprecipitation (CLIP) against both transcripts within one injury model, together with single‐nucleotide m^6^A mapping on endogenous cardiac transcripts.

### 4.2. Emerging m^6^A‐Associated Readers and Non‐m^6^A Epitranscriptomic Pathways

Beyond the core YTHDF pathways, current evidence for additional epitranscriptomic regulators of FTH1 in the heart remains fragmentary, with most candidate m^6^A‐associated RBPs linked to FTH1 through indirect mechanisms or noncardiac precedents. In hypoxia–reoxygenation, hnRNPA2B1 stabilizes m^6^A‐modified *Pfn2* mRNA, and elevated PFN2 in turn suppresses FTH1 protein and promotes ferroptosis during myocardial I/R injury [24]. In Angiotensin II (Ang II)–exposed AC16 cells, HNRNPC knockdown prevents a late fall in FTH1 protein [25]. However, the molecular intermediaries between HNRNPC and FTH1 remain undefined, and the evidence currently rests on correlative protein‐level data rather than direct binding assays. In cervical cancer cells, Li et al. [26] used methylated RNA immunoprecipitation (MeRIP)–qPCR to demonstrate METTL14‐dependent m^6^A modification of *FTH1* mRNA leading to its degradation. Similarly, IGF2BP1 has been shown to stabilize *FTH1* mRNA in ovarian cancer cells [27], but its role in cardiac FTH1 regulation has not been examined. Resolving this question will require single‐nucleotide‐resolution m^6^A mapping on endogenous *FTH1* transcripts in primary cardiomyocytes.

Beyond m^6^A, other RNA modifications are emerging as regulators of FTH1. NSUN5‐mediated 5‐methylcytosine (m^5^C) sites in the *FTH1* 5 ^′^‐UTR and the *FTL* 3 ^′^‐UTR have been mapped by bisulfite sequencing at single‐nucleotide resolution in rat mesenchymal stem cells, where recruitment of the mitochondrial chaperone TRAP1 raises FTH1 and FTL protein levels, thereby suppressing ferroptosis [28]. Whether this m^5^C/NSUN5/TRAP1 pathway operates in cardiomyocytes remains to be determined, but it indicates that *FTH1* mRNA stability is governed by a broader epitranscriptomic code beyond m^6^A alone.

### 4.3. Classical RBPs: Context‐Dependent Effects on Cardiac Ferroptosis

In addition to the m^6^A‐associated readers discussed above, several classical RBPs may influence FTH1 abundance indirectly, primarily through the autophagy–ferroptosis axis rather than through direct transcript‐specific regulation. ELAVL1 (ELAV‐like RNA‐binding Protein 1, also known as HuR) acts on FTH1 in both directions. It promotes autophagy‐dependent ferroptosis by stabilizing *BECN1* mRNA, which activates ferritinophagy [29]. ELAVL1 upregulation has been confirmed in cardiac I/R downstream of FOXC1 [30], though the full ELAVL1/BECN1/ferritinophagy chain has not been reconstituted in cardiomyocytes. However, ELAVL1 can also suppress ferroptosis: In DOX cardiotoxicity, HuR stabilizes FTO‐demethylated *p53/p21/Nrf2* transcripts [31]. This duality, proferroptotic via BECN1 stabilization and antiferroptotic via Nrf2 stabilization, mirrors the YTHDF2 paradox discussed in Section 4.1.3 and illustrates a general principle: RBPs that act on multiple transcripts simultaneously can produce context‐dependent, even opposing, effects on ferroptosis depending on which target predominates.

### 4.4. NcRNA‐Mediated Posttranscriptional Regulation of FTH1

NcRNAs have also been implicated in FTH1 regulation during cardiac ferroptosis, though only a subset operate through direct posttranscriptional mechanisms rather than transcription factor–mediated pathways.

The most direct evidence for miRNA‐mediated posttranscriptional repression of FTH1 in the heart comes from Zheng et al. [32], who identified circSnx12 as a competitive endogenous RNA (ceRNA) sponge for miR‐224‐5p in a pressure overload model of heart failure. miR‐224‐5p directly targets the *Fth1* 3 ^′^‐UTR, as confirmed by a wild‐type versus mutant luciferase reporter in HL‐1 atrial cells [32]. In transverse aortic constriction (TAC) myocardium, *Fth1* transcript and protein fell in parallel [32] (concordant regulation, Section 2.3).

The two steps of the axis rest on different evidence. The miR‐224‐5p–*Fth1* interaction is supported by the reporter assay; the upstream circSnx12–miR‐224‐5p step is inferred, since circSnx12 itself was neither overexpressed nor silenced and no AGO2 immunoprecipitation or RNA pull‐down was performed [32]. As with other proposed ceRNA circuits, stoichiometric sufficiency has not been established: Whether the circRNA is abundant enough relative to the miRNA pool to act as a competitive sink in vivo remains unknown.

This axis raises a question specific to FTH1. *FTH1* mRNA contains an IRE in its 5 ^′^‐UTR (bound by IRPs) and is now shown to be simultaneously targeted at its 3 ^′^‐UTR by a miRNA, raising the possibility that these two regulatory elements could operate competitively or cooperatively. However, no study to date has examined whether IRP occupancy at the 5 ^′^‐IRE influences miR‐224‐5p accessibility at the 3 ^′^‐UTR, or vice versa. Determining whether these two cis‐regulatory systems interact physically or function independently represents an important mechanistic question for future work (Table 1, Layer 2 entries).

## 5. NCOA4‐Mediated Ferritinophagy: The Proteolytic Fate of FTH1

Even when translational repression (Section 3) and mRNA stabilization (Section 4) cooperate to sustain FTH1 protein output, the ferritin pool remains subject to a third and final layer of control: selective autophagic degradation, termed ferritinophagy. NCOA4 was identified as the selective cargo receptor for ferritin [3, 7, 33]. Two independent studies demonstrated that NCOA4‐dependent ferritinophagy is activated during ferroptosis induction: Depletion of NCOA4 or autophagy components suppresses ferritin degradation, reduces the LIP, and confers ferroptosis resistance [53, 54]. Ferritinophagy is therefore a decisive step that converts the protective iron‐sequestering function of FTH1 into a proferroptotic iron source.

Recognition depends on FTH1 but is exerted on the whole cage. NCOA4 engages the conserved surface arginine R23, which is present on FTH1 and absent from FTL, and recombinant FTL‐only cages are not bound [34]. Ferritin complexes labeled with an R23A mutant subunit nonetheless still reach lysosomal puncta, indicating that one or a few wild‐type FTH1 subunits within a 24‐mer suffice to target the entire complex [34]. The cargo delivered to the phagophore is therefore the intact heteropolymer, and associated FTL subunits are degraded with it, as direct imaging of FTH1 and FTL corecruitment into autophagosomes confirms [14]. For the heart, the practical consequence is that ferritinophagy depletes total ferritin rather than the H subunit selectively, so ferroxidase capacity and storage capacity are lost together. FTL was not measured in any cardiac study reviewed here, so whether this holds in the heart is untested (Table 1).

### 5.1. Regulatory Networks Governing NCOA4‐Mediated FTH1 Degradation

The rate of ferritinophagy is set by NCOA4 abundance, which is itself controlled by iron sensing, posttranslational modification, and inputs from the other regulatory layers.

#### 5.1.1. Iron‐Dependent Feedback via HERC2

Under iron‐replete conditions, the E3 ubiquitin ligase HERC2 ubiquitinates NCOA4 for proteasomal degradation; when iron is scarce, NCOA4 escapes turnover and accumulates, amplifying ferritinophagy [34]. This creates a self‐reinforcing loop: Iron depletion stabilizes NCOA4, which degrades ferritin and releases iron, partially restoring the LIP. The circuit is analogous to the IRP/IRE system but operates at the protein level.

Pressure overload increases mitochondrial iron consumption, and this circuit may invert as a result. If iron released from ferritin is taken up by mitochondria rather than replenishing the cytosolic LIP, cytosolic iron remains low, HERC2‐mediated NCOA4 degradation is not triggered, and ferritin depletion becomes self‐amplifying. The *Ncoa4* knockout time course is consistent with this [8], and mitochondrial uptake of ferritin‐derived iron is the step that would test it.

#### 5.1.2. Posttranslational Modifications of NCOA4

Beyond HERC2‐mediated degradation, NCOA4 activity is fine‐tuned by at least two posttranslational modifications with potential cardiac relevance. The DNA damage‐responsive kinase ATM (ataxia‐telangiectasia mutated) phosphorylates NCOA4 at S550, enhancing the NCOA4–FTH1 interaction and accelerating ferritinophagy independently of p53 [35]. ATM is itself activated by I/R‐induced DNA damage in the heart, although the phosphorylation has been demonstrated only in mouse embryonic fibroblasts (MEFs) and tumor cell models.

SUMOylation acts in the opposite direction. Xue et al. [36] showed that SUMO1 modification of NCOA4 at K81, K343, and K600 recruits the deubiquitinase OTUB1, which strips NCOA4 of its ubiquitin chains and stabilizes the protein. The SUMO protease SENP2 reverses this by de‐SUMOylating NCOA4 and returning it to ubiquitin‐dependent degradation. SENP2 expression declines during I/R, resulting in the accumulation of SUMOylated NCOA4, ultimately amplifying ferritinophagy and cardiomyocyte ferroptosis [36]. The *Senp2* knockout in that study was global rather than cardiac‐restricted, and NCOA4 itself was never knocked down (Table 1).

#### 5.1.3. Cross‐Layer Integration: m^6^A and NRF2

Two of the inputs to NCOA4 come from outside ferritinophagy itself. *NCOA4* mRNA is itself a substrate of METTL3/YTHDF2‐mediated m^6^A decay (Section 4.1.3) [23], so Layer 2 sets the abundance of the receptor that determines the rate of Layer 3. NRF2 spans a wider range: It promotes *FTH1* transcription (Section 2.3), and it also sustains HERC2‐dependent NCOA4 turnover and VAMP8‐dependent autophagic flux [14]. Loss of NRF2 therefore raises ferritin protein while disabling its delivery to lysosomes, the apoferritin‐trapping phenotype described in Section 2.4.

Several cardiac ncRNA pathways converge on this node during I/R injury. miR‐155‐5p carried by cardiac extracellular vesicles directly represses *Nfe2l2* and lowers *Fth1* transcript and protein [55], and loss of the protective lncRNA *Oip5-as1* suppresses p62/KEAP1/NRF2 signaling and reduces FTH1 protein [56]. Pharmacological NRF2 activation attenuates cardiac ferroptosis in multiple preclinical models [15].

NRF2 is therefore the one regulator whose perturbation acts on two layers at once. Activation raises *FTH1* transcription and suppresses NCOA4, but it also restores the fusion step that completes ferritin degradation, so the net effect on FTH1 protein is not predictable from any one arm alone (Section 6.2).

### 5.2. Ferritinophagy‐Driven FTH1 Depletion Across Cardiac Ferroptosis Contexts

Ferritinophagy has now been documented across the major categories of cardiac ferroptosis, with distinct upstream signals converging on NCOA4 as a common effector.

#### 5.2.1. Pressure Overload Heart Failure

The most genetically rigorous evidence for ferritinophagy as a causal driver of cardiac FTH1 depletion comes from Ito et al. [8], who employed cardiac‐specific *Ncoa4* knockout mice subjected to TAC, with all in vitro work performed in isolated adult mouse ventricular cardiomyocytes. In NCOA4‐competent hearts, FTH1 protein declined from postoperative Day 5, whereas *Fth1* transcript rose at Days 5 and 7, accompanied by LIP expansion and ferroptosis marker elevation. Cardiac‐specific *Ncoa4* deletion preserved FTH1 protein abundance, reduced the LIP, and attenuated both ferroptosis and cardiac dysfunction [8]. The upstream signal driving NCOA4 activation in this model appears to be the progressive iron depletion itself: As pressure overload increases myocardial energy demand and mitochondrial iron consumption, the resulting decline in cytosolic iron would relieve HERC2‐mediated NCOA4 degradation (Section 5.1), initiating a self‐amplifying cycle of ferritinophagy.

#### 5.2.2. Myocardial I/R Injury

I/R injury engages ferritinophagy through at least two temporally distinct mechanisms. During reperfusion, Fe^2+^ accumulation and NCOA4 upregulation coincide with FTH1 protein decline [57]. Additionally, SENP2 expression declines during reperfusion, leading to NCOA4 SUMOylation accumulation that enhances OTUB1‐mediated NCOA4 protein stabilization and amplifies ferritinophagy (Section 5.1.2) [36].

NCOA4 upregulation during reperfusion coincides temporally with the frataxin/IRP1‐mediated translational blockade described in Section 3.3.1: FTH1 synthesis is suppressed, and its degradation is accelerated at the same time. I/R is the one setting where two layers have been shown to act on FTH1 concurrently, and lactate‐driven YTHDF2 activation may add a third (Section 6.1).

#### 5.2.3. Sepsis‐Induced Cardiac Injury

Lipopolysaccharide (LPS)‐induced septic cardiomyopathy represents a distinct pathological context in which systemic inflammation directly activates NCOA4‐mediated ferritinophagy. Li et al. [39] demonstrated that LPS increases NCOA4 expression and intracellular Fe^2+^ levels while decreasing ferritin protein in both in vivo and in vitro models. Iron released by NCOA4‐mediated degradation is transported into mitochondria via SFXN1, triggering mitochondrial ROS generation and ferroptosis, and both ferrostatin‐1 and dexrazoxane attenuated this cascade in vivo [39].

This ferritinophagy‐to‐mitochondrial axis is mechanistically distinct from the iron‐sensing (HERC2) and posttranslational (SENP2) pathways operative in I/R. The molecular signal linking inflammation to NCOA4 remains unidentified, and the axis rests on a single study.

#### 5.2.4. Metabolic Stress‐Associated Cardiac Injury

Metabolic stress activates NCOA4‐mediated ferritinophagy through at least two distinct upstream pathways. In obesity‐associated cardiac injury, Zhu et al. [37] identified IL‐6/STAT3 signaling as a transcriptional activator of NCOA4: High‐fat diet feeding activated STAT3, which directly upregulated NCOA4, leading to ferritinophagy‐mediated FTH1 depletion and iron overload. Pharmacological STAT3 inhibition with piperlongumine restored FTH1 protein levels and attenuated cardiac ferroptosis, whereas STAT3 overexpression exacerbated the phenotype [37]. This is also the one cardiac study that measured ferritinophagic flux directly rather than inferring it from steady‐state abundance (Section 5.3). In diabetes‐associated myocardial I/R injury, DNA Methyltransferase 1 (DNMT1)–mediated DNA hypermethylation has been implicated in NCOA4 upregulation, and DNMT1 inhibition alleviated ferroptosis [38]. That study measured NCOA4 and ferritin abundance only, without any assay of autophagic flux (Table 1).

#### 5.2.5. Synthesis: Convergent Effector and Divergent Upstream Signals

The upstream triggers diverge across contexts: iron‐sensing feedback in pressure overload, posttranslational modification of NCOA4 in I/R, inflammatory signaling in sepsis, inflammatory transcription in obesity, epigenetic reprogramming in diabetes, and environmental particulate exposure [40]. All converge on NCOA4 as the effector that executes FTH1 degradation (Table 1).

Ferritinophagy thus represents the terminal degradative checkpoint for FTH1 protein. Even when IRP/IRE suppression is partially relieved or *FTH1* mRNA is maintained, excessive NCOA4‐dependent ferritinophagy can override by depleting the protein pool that already exists(Figure 1) [58, 59]. NCOA4 is correspondingly the one node whose inhibition attenuates FTH1 loss regardless of the initiating stimulus, as the cardiac‐specific *Ncoa4* knockout demonstrates [8]. The same redundancy that makes NCOA4 attractive as a target also means that suppressing any single upstream input is unlikely to be sufficient.

### 5.3. How Much of This Is Flux?

Ferritinophagy is by definition a flux, and steady‐state abundance of NCOA4, LC3‐II, p62, or FTH1 cannot establish it. Exactly one cardiac study applied a lysosomal or autophagic inhibitor clamp directly to the FTH1 readout, in the obesity model, where 3‐methyladenine (3‐MA) or bafilomycin A1 pretreatment restored FTH1 protein, supported by an mRFP‐GFP‐LC3B tandem reporter [37]. Three rest on partial evidence, and two on steady‐state abundance alone, with the specific assay recorded for each in Table 1. For future cardiac work, the minimum standard is an inhibitor clamp applied to the ferritin readout itself, ideally with an orthogonal turnover measurement such as a tandem fluorescent reporter or NCOA4–FTH1 coimmunoprecipitation.

## 6. Integrative Perspective and Therapeutic Implications

### 6.1. Layer Convergence and the Attribution of FTH1 Loss

Iron depletion is the one upstream signal known to act on more than one layer at once. Falling cytosolic iron both activates IRP‐mediated translational repression (Section 3) and stabilizes NCOA4 by relieving HERC2‐dependent degradation (Section 5.1.1), so a single change suppresses FTH1 synthesis and accelerates its degradation simultaneously. NRF2 spans a comparable range at the level of the regulators themselves (Section 5.1.4).

Reperfusion is where this convergence should be most visible. Frataxin decline activates the IRP switch and suppresses new *Fth1* translation (Section 3.3.1), whereas NCOA4 upregulation and SENP2‐mediated de‐SUMOylation accelerate degradation of the existing protein pool (Section 5.2.2). Lactate‐driven YTHDF2 activation would add *Fth1* mRNA decay to the same window (Section 4.1.1), placing three layers on one protein within hours of each other.

Since the layers converge, the pattern of change rather than the magnitude identifies which one is responsible. Table 2 sets out which observations are consistent with each layer and which additional measurement resolves the remainder. Two features of that logic are worth stating. Transcript direction separates Layer 2 from Layers 1 and 3; separating Layer 1 from Layer 3 requires a lysosomal clamp or a polysome shift, since translational repression and ferritinophagy both leave the transcript intact. A rise in FTH1 protein requires an iron measurement to interpret, because apoferritin trapped in unfused autophagosomes registers as increased protein whereas the LIP expands.

**Table 2:** Attributing a change in FTH1 protein to a regulatory layer.

Observation	Consistent with	Resolving measurement	Cardiac exemplar
Protein ↓, transcript unchanged or ↑	Layer 1 or Layer 3	Lysosomal or autophagic clamp (Layer 3); polysome or ribosome profiling (Layer 1)	Ito, *Ncoa4* cKO [8]
Protein ↓, transcript ↓	Layer 2, or transcriptional input	Transcription chase to separate decay from reduced synthesis; BACH1/NRF2 status	Xiang, lactate–YTHDF2 [10]
Protein ↑, transcript unchanged	Layer 1 relieved	IRE‐binding assay	Yin, ALKBH5/YTHDF1 [2]
Protein restored by lysosomal or autophagic inhibition	Layer 3	This is the direct test	Zhu, STAT3–NCOA4 [35]
Protein ↑ but LIP also expanded	Ferritin delivery blocked downstream of cargo capture	Ferritin iron loading; autophagosome–lysosome fusion	Anandhan, NRF2/VAMP8 [36]

*Note:* Layer 1, IRP/IRE‐mediated translational repression (Section 3); Layer 2, epitranscriptomic and RNA‐binding protein control of transcript fate (Section 4); Layer 3, NCOA4‐mediated ferritinophagy (Section 5). Transcript direction separates Layer 2 from Layers 1 and 3, but does not separate Layer 1 from Layer 3: Translational repression and ferritinophagy both leave the transcript intact, so only a lysosomal clamp or a polysome shift discriminates between them. A rise in FTH1 protein is not self‐interpreting. Apoferritin trapped in unfused autophagosomes registers as increased protein, whereas the labile iron pool expands, which is why the final row requires an iron measurement rather than an immunoblot. Exemplars cite the cardiac study in which the pattern was observed, except for the final row, which has no cardiac exemplar and is drawn from a noncardiac system.

No cardiac study has measured all three on the same FTH1 pool in one injury model. A single reperfusion time course would show whether the layers act sequentially or in parallel and whether intervention at one node can be sufficient (Section 6.2).

### 6.2. Therapeutic Strategies Targeting FTH1 Regulatory Checkpoints

The strategies below fall into three tiers by their relationship to FTH1 protein fate. Downstream agents (Section 6.2.1) limit ferroptotic damage without restoring FTH1 and are indifferent to which layer is responsible. Layer‐directed agents (Sections 6.2.2, 6.2.3, and 6.2.5) are aimed at restoring FTH1 abundance and therefore require the attribution set out in Table 2. Regulators with divergent targets (Section 6.2.4), notably YTHDF2, NRF2, and ELAVL1, act on more than one determinant at once, so their net effect depends on the prevailing transcript landscape.

Both IRP‐directed and NCOA4‐directed strategies are aimed at raising FTH1, and both can overshoot. Cardiomyocyte‐specific Irp1/Irp2 double deletion raised ferritin H protein to ~249% of control yet produced cardiac iron deficiency, impaired mitochondrial respiration, and loss of contractile reserve [21]. Preserved or elevated FTH1 is therefore not a therapeutic endpoint in itself; the endpoint is an LIP held within a range that neither drives Fenton chemistry nor starves iron–sulfur and heme biosynthesis. Layer‐directed interventions should accordingly be evaluated against iron‐supply readouts (mitochondrial iron, Complex I activity, and contractile reserve) rather than against FTH1 abundance alone.

#### 6.2.1. Iron Chelation and Ferroptosis Inhibition

Deferoxamine and deferiprone reduce the LIP, thereby directly limiting the substrate for Fenton‐mediated lipid peroxidation [1, 60]. By lowering intracellular iron, these chelators also shift the IRP/IRE equilibrium toward enhanced IRP binding, sustaining *FTH1* translational repression. This is the physiologically appropriate response to low‐iron conditions rather than pathological FTH1 suppression. Iron chelation thus protects cardiomyocytes primarily by reducing iron toxicity, not by restoring FTH1 protein levels.

Ferrostatin‐1 and liproxstatin‐1 interrupt ferroptosis downstream of iron overload by scavenging lipid peroxyl radicals [61, 62]. Flavonoids such as baicalein and luteolin reduce infarct size in rat I/R through the same downstream route, without reported effect on NCOA4 or ferritinophagy [58]. However, these agents target the consequences rather than the causes of FTH1 depletion and do not address the multilevel dysregulation that drives ferritin loss.

#### 6.2.2. Modulating the IRP/IRE Axis

Promoting FBXL5‐mediated IRP2 degradation could relieve the translational blockade under pathological iron fluctuations [20]. The identification of cardiac‐specific IRP2 regulators, USP38 as a stabilizer [17] and TRIM28 as a destabilizer [18], opens the possibility of tissue‐targeted IRP2 modulation. Additionally, restoring ISC biosynthesis through frataxin supplementation has been shown to reactivate IRP1 aconitase function and reduce infarct size in a murine I/R model [16], suggesting that targeting the ISC/IRP1 switch may represent an alternative entry point. No IRP2‐targeted agent currently exists, and any that emerges will need a therapeutic window defined against cardiomyocyte iron rather than against FTH1 protein alone.

#### 6.2.3. Inhibiting NCOA4‐Mediated Ferritinophagy

Direct NCOA4 inhibitors are not available, so the cardiac evidence rests on upstream suppression and genetic proof of principle. ATM inhibitors may reduce NCOA4 S550 phosphorylation [35], though their pleiotropic effects on DNA damage response necessitate caution. Piperlongumine inhibits STAT3‐driven NCOA4 upregulation with demonstrated cardioprotective effects in obesity‐associated cardiomyopathy [37]. The strongest support is genetic: Cardiomyocyte‐specific *Ncoa4* knockout preserved FTH1 and attenuated both ferroptosis and dysfunction under pressure overload [8], establishing ferritinophagy inhibition as a viable target even though no pharmacological equivalent exists.

#### 6.2.4. NRF2 Activation as a Multilayer Intervention

NRF2 acts at three points relevant to FTH1 (Section 5.1.4), and pharmacological activators are already in clinical use: sulforaphane, dimethyl fumarate, and bardoxolone methyl [15]. The arms do not all point the same way. Raising *FTH1* transcription and suppressing NCOA4 both preserve ferritin, but restoring VAMP8‐dependent autophagosome–lysosome fusion permits ferritin already captured by NCOA4 to be degraded. Whether NRF2 activation raises or lowers FTH1 protein in a given cardiac setting therefore depends on which arm dominates, and no cardiac study has measured the three separately. The same caution applies to any regulator with divergent targets, including YTHDF2 (Section 4.1.3) and ELAVL1 (Section 4.3).

#### 6.2.5. Emerging RNA‐Based Concepts

Emerging concepts include engineered IRE decoys to sequester IRPs and relieve *FTH1* translational repression, enhancement of the ALKBH5/YTHDF1 translational axis (Section 4.1.2) to promote FTH1 protein output [2], and NCOA4‐targeting siRNAs and miRNA modulators such as anti‐miR‐224‐5p (Section 4.4). Delivery to cardiomyocytes via cardiotropic lipid nanoparticles or AAV9 vectors is an active area of development [63], but none of these *FTH1*‐directed RNA strategies has yet been validated in cardiac models.

### 6.3. Open Questions and Future Directions

Within‐experiment measurement is itself rare. Across the cardiac studies surveyed, FTH1 mRNA and protein were assayed in the same experiment in only five. The two mechanisms acting on translation or on the assembled protein produced discordance: the TAC/Ncoa4 study [8] and the ALKBH5 study [2]. The remaining three found transcript and protein concordant. The framework is therefore proposed as an organizing scheme for a literature in which mRNA and protein are seldom measured together, not as a claim that discordance is the usual cardiac finding.

A central unresolved issue is the cell‐type‐specific and temporal heterogeneity of FTH1 regulation. The relative contribution of each regulatory layer likely varies across cardiac cell types (cardiomyocytes, fibroblasts, endothelial cells, and macrophages) and across disease stages. At present, this is essentially unexamined, since almost all cardiac FTH1 data derive from cardiomyocytes or from whole‐heart homogenate in which cell‐type contributions cannot be separated.

A structural limitation of the current evidence base is its dependence on immortalized cell lines. Of the cardiac studies surveyed, mechanistic work on FTH1 was performed predominantly in H9c2 cardiomyoblasts, the AC16 human cardiomyocyte line, and HL‐1 atrial cells. Adult ventricular cardiomyocytes were used for FTH1‐directed experiments in only three studies [8, 19, 21]. No cardiac study of epitranscriptomic FTH1 regulation has used adult cardiomyocytes, human‐induced pluripotent stem cell–derived cardiomyocytes, or human myocardium. This matters because adult cardiomyocytes are postmitotic, have far higher mitochondrial iron demand than proliferating lines, and engage different dominant cell death programs.

Compartment is a second unaddressed variable: The USP38/IRP2 axis was established exclusively in left atrial tissue and an atrial‐derived cell line [17], and its ventricular relevance is unknown, whereas essentially all other cardiac FTH1 data are ventricular. Furthermore, no study in this review reported FTH1 mRNA or protein in human myocardium, let alone both; the nearest approaches measured nonheme iron, IRE‐binding activity, and TFR1, but not ferritin [21, 18]. This is the single largest gap in the framework.

Equally unresolved are the temporal dynamics: The sequential or parallel activation of the three layers has not been dissected within a single cardiac injury model. In particular, how m^6^A marks on FTH1 evolve across the I/R continuum, and whether they interact with the simultaneous IRP/IRE response, remains uninvestigated [12]. Without the single‐nucleotide mapping discussed in Section 4.1.3, it also remains unclear whether the m^6^A sites recognized by YTHDF2 (promoting degradation [12]) overlap with those recognized by YTHDF1 (promoting translation [2]). Single‐cell and spatial multiomics profiling, combined with time‐resolved proteomic and epitranscriptomic analyses during controlled I/R, will be needed to resolve this heterogeneity [64].

Addressing these questions will require methodological advances that are currently lacking. No cardiac study to date has mapped m^6^A sites on *FTH1* mRNA at single‐nucleotide resolution; existing evidence relies on MeRIP‐qPCR or RIP, which confirm gene‐level methylation or reader binding but cannot pinpoint which adenosines are modified. Without site‐specific information, it remains unclear whether the m^6^A sites recognized by YTHDF2 (promoting degradation [12]) overlap with those recognized by YTHDF1 (promoting translation [2]), or whether distinct modification patterns route *FTH1* mRNA toward divergent fates. Furthermore, m^6^A modification can alter local mRNA secondary structure through the “m^6^A switch” mechanism, potentially exposing or masking binding sites for RBPs such as HuR. Whether such structural remodeling occurs on *FTH1* mRNA remains an open question.

Beyond these priorities, the current three‐layer framework likely underestimates the full complexity of FTH1 regulation. For example, compensatory roles of mitochondrial ferritin and noncanonical NCOA4 modifications (O‐GlcNAcylation, methylation, and acetylation) [65] remain unexplored in cardiac systems. More fundamentally, several mechanisms highlighted here still lack direct evidence in cardiomyocytes or cardiac tissue: ISC‐dependent IRP2 sensing (established in cancer cells [20]), ATM‐dependent NCOA4 phosphorylation (established in MEFs [35]), and IGF2BP1‐mediated FTH1 stabilization (established in ovarian cancer [27]). Direct testing in cardiac systems will be necessary to determine how broadly this model applies to cardiac ferroptosis contexts [15].

## 7. Conclusions

FTH1 protein abundance in the diseased heart is set by three regulatory layers acting downstream of transcription: IRP/IRE translational repression, epitranscriptomic and RBP control of transcript fate, and NCOA4‐mediated ferritinophagy. Treating them as one framework makes two things visible. First, the pattern of change across transcript and protein identifies which layer is responsible, and each layer implies a different intervention (Table 2). Second, since FTH1 protein reports iron‐buffering capacity only indirectly, raising it is not by itself a therapeutic endpoint: Complete derepression of IRP signaling raises ferritin while producing cardiac iron deficiency [21].

## Author Contributions

Conceptualization: F.Z. and J‐B.Z.; literature search and data curation: F.Z., J‐B.Z., and Y‐Q.Y.; writing original draft preparation: F.Z. and J‐B.Z.; writing review and editing: R‐X.W., T‐P.W., and D.W.; visualization: F.Z. and Y‐F.Y.; supervision: R‐X.W.; funding acquisition: R‐X.W. F.Z., J‐B.Z., and Y‐Q.Y. contributed equally to this work.

## Funding

This work was supported by the National Natural Science Foundation of China (10.13039/501100001809, 82370342) and the Natural Science Foundation of Jiangsu Province (10.13039/501100004608, BK20231145).

## Disclosure

All authors have read and agreed to the published version of the manuscript.

## Ethics Statement

The authors have nothing to report.

## Consent

The authors have nothing to report.

## Conflicts of Interest

The authors declare no conflicts of interest.

## Data Availability

Data sharing is not applicable to this article as no datasets were generated or analyzed during the current study.
